# Drug-related emergency department visits in older patients: an applicability and reliability study of an existing assessment tool

**DOI:** 10.1007/s11096-022-01456-x

**Published:** 2022-07-15

**Authors:** Thomas Gerardus Hendrik Kempen, Anton Hedman, Ulrika Gillespie

**Affiliations:** 1 Department of Pharmacy, Uppsala University, Uppsala, Sweden; 2 Primary Care and Health, Uppsala County Council, Uppsala, Sweden; 3 Hospital Pharmacy Department, Uppsala University Hospital, Uppsala, Sweden; 4 Nivel, Netherlands Institute for Health Services Research, Utrecht, the Netherlands

**Keywords:** Drug therapy, Emergency service, Health care, Hospital, Outcome assessment, Patient harm, Quality of health care, Reliability of results

## Abstract

**Background:**

AT-HARM10 is a research tool to identify possible drug-related hospital admissions. It is unclear whether the tool can be applied to emergency department visits as well.

**Aim:**

The aim of this study was to investigate the applicability and reliability to identify drug-related emergency department visits in older patients with AT-HARM10.

**Method:**

A random sample of 400 patients aged 65 years or older from a clinical trial in four Swedish hospitals was selected. All patients’ emergency department visits within 12 months after discharge were assessed with AT-HARM10. The main outcome measures were the percentage of successfully assessed visits for applicability and the interrater reliability (Cohen’s kappa).

**Results:**

Of the initial sample (n = 400), 113 patients [median age (interquartile range): 81 (76–88) years] had at least one emergency department visit within 12 months. The patients had in total 184 visits, of which 179 (97%) were successfully assessed. Fifty-three visits (29%) were possibly drug-related. The Cohen’s kappa value was 0.70 (substantial).

**Conclusion:**

It seems applicable and reliable to identify possible drug-related emergency department visits in addition to hospital admissions in older patients with AT-HARM10. As a consequence, the tool has been updated to support its novel use in clinical research.

**Supplementary Information:**

The online version contains supplementary material available at 10.1007/s11096-022-01456-x.

## Impact statements

- In addition to hospital admissions, AT-HARM10 can be used in research as a tool to identify possible drug-related emergency department visits
- The lack of a need for a clinical expert panel for the use of AT-HARM10 makes it a cost-saving method in relation to other methods to identify drug-related emergency department visits
- An updated version of this tool was made available as a consequence of this study to support its novel use in clinical research

## Introduction

Pharmacotherapy is perhaps the most frequently used treatment for medical conditions. Unfortunately, it can also do harm, putting a burden on patients and the healthcare system [1, 2]. Older patients’ hospital admissions and emergency department (ED) visits are frequently caused by drug-related problems (DRPs) [3–7], defined as an event or circumstance involving drug therapy that actually or potentially interferes with desired health outcomes [8]. Two recent systematic reviews on drug-related hospital admissions (DRAs) report an average prevalence of 15% and 21% of which at least a third seems preventable [3, 9]. DRAs and drug-related ED visits are important outcomes in research areas with a strong link to pharmacotherapy, such as clinical pharmacy [10, 11]. However, great heterogeneity in methods to measure such visits exists and validated methods are lacking [3, 5, 11]. Most tools that have been validated, e.g., the Naranjo algorithm and Hallas criteria, have not been developed to identify the full range of DRPs that can cause hospital admissions [12–15]. Methods often include assessment by an expert panel of experienced clinicians, making it time-consuming and costly. In 2018, a standardized chart review method was published, the DRA adjudication guide [16]. The guide was validated in terms of feasibility and interrater reliability (IRR), but its accuracy (i.e. sensitivity and specificity) is unknown and it also requires the use of an expert panel. We therefore developed and validated a practical tool to identify possible DRAs in older patients, the Assessment Tool to identify Hospital Admissions Related to Medications (AT-HARM10) [17]. AT-HARM10 consists of ten yes–no questions to distinguish between admissions that are unlikely to be and those that are possibly drug-related. The tool was validated for use by a pair of final-year pharmacy students in comparison to expert panel assessments, resulting in a moderate to substantial IRR (kappa values between 0.45 and 0.75) and accuracy measurements between 70 and 86% [17]. Methods to identify DRAs are not necessarily applicable to ED visits because of lower availability of diagnostic data and other information [5, 18]. It is therefore relevant to investigate whether AT-HARM10 can be applied to ED visits in addition to hospital admissions.

## Aim

The aim of this study was to investigate the applicability and reliability to identify drug-related ED visits in older patients with AT-HARM10.

## Ethics approval

This study was part of the MedBridge project that has received ethical approval from the Swedish Central Ethical Review Board (CEPN; registration number: Ö21-2016). No additional ethical approval was needed in accordance with Swedish legislation that only requires new approval in case of significant changes that may affect the initial benefit-risk assessment.

## Method

### Study design, setting and population

This was an applicability and reliability study of AT-HARM10 that was part of the MedBridge trial of which the protocol and main results have been published elsewhere [19, 20]. The trial was conducted from February 2017 until October 2018 at four hospitals in Sweden: Uppsala University Hospital, and the hospitals in Enköping, Gävle and Västerås. Two wards per hospital were included. These wards were either a general internal medicine or an internal medicine subspecialty ward. Patients aged 65 years or older that were admitted to one of the participating wards for at least 1 day were included. Patients were excluded if they had undergone a medication review by a clinical pharmacist within the last month, did not reside in the hospital’s county, or were receiving palliative treatment. The trial included 2637 patients that had in total 2167 ED visits during 12 months follow-up (1039 (39%) patients had at least one ED visit) [19]. For the present study, a sample of 400 patients were randomly selected from all MedBridge trial participants with Microsoft Excel, stratified per hospital, aiming for at least 100 patients with an ED visit. No formal sample size calculation was made. All patients with at least one ED visit within 12 months after hospital discharge were included.

### Data collection, outcome measures and analysis

Baseline and ED visit data within 12 months after hospital discharge were extracted from the counties’ electronic health record (EHR) systems and healthcare registries. Only separate ED visits were of interest in this study: ED visits that were directly (within four hours) followed by a hospital admission, were considered part of the admission. A pair of researchers assessed all participants’ ED visits with AT-HARM10 [17] between September 2020 and January 2021. An ED visit was considered drug-related if a DRP was either the main reason for the visit or a significantly contributing reason (i.e., without the DRP, the patient would not have been admitted). The physician notes from the ED visit, medication list and any laboratory data available from the ED visit in the patients’ EHR system were used for the assessments. First, the researchers independently assessed each visit, classifying it as either unlikely or possibly drug-related. The researchers then discussed the visits they disagreed on to reach consensus. An experienced clinical pharmacist and researcher (TK) was available in case of doubt. The main outcome measures were the percentage of successfully assessed ED visits and the IRR. Successfully assessed ED visits were defined as ED visits for which the pair of researchers were able to decide whether the visit was unlikely or possibly drug-related. For IRR, Cohen’s kappa value before consensus discussion was calculated and interpreted following the method by Charles Zaiontz [21]. All analyses and descriptive statistics were performed with Microsoft Excel.

## Results

Of the 400 participants that were randomly selected, 113 had at least one ED visit and were included (Table 1). The median age (interquartile range) of these patients was 81 years (76–88) and a median of 9 (interquartile range 7–13) drugs were prescribed.

**Table 1:** Population baseline characteristics of assessed patients (at least one ED visit)

Characteristic	Patients assessed (n = 113)
Age, median years (IQR)	81 (76–88)
Female sex, % (number)	50 (57)
Medications, median number (IQR)	9 (7–13)
ED visits, median number (IQR)	1 (1–2)
Hospital, % (number)	
Enköping	36
Gävle	25
Uppsala	30
Västerås	22

*ED* emergency department, *IQR* interquartile range

The patients had in total 184 ED visits. Two researchers, one final-year pharmacy student and one recent graduate (AH), assessed the visits after having received a training session, including test cases and case discussions, by two clinical pharmacists and researchers that initially developed AT-HARM10 (TK and UG). Of all 184 visits, 97.3% (n = 179) were successfully assessed (Table 2). Three visits were not assessed, because it concerned planned visits that were part of treatment follow-up. Two visits could not be assessed, because written notes were missing in the EHR. After consensus discussion, 53 visits (28.8%) were identified as possibly drug-related. For 87.2% (n = 156) successfully assessed visits, there was initial agreement between the researchers, corresponding to a Cohen’s kappa of 0.70 (substantial).

**Table 2:** Applicability and interrater reliability study results

Outcome	ED visits (n = 184), % (number)
Successfully assessed	97.3 (179)
Unlikely drug-related	68.5 (126)
Possibly drug-related	28.8 (53)
Unsuccessfully assessed	2.7 (5)
Planned visit	1.6 (3)
Missing written notes in EHR	1.1 (2)
Initial agreement between researchers	87.2^a^ (156)
Corresponding Cohen’s kappa value (interpretation)	0.70 (substantial)

*ED* emergency department, *EHR* electronic health record

^a^Percentage of 179 successfully assessed visits

## Discussion

### Statement of key findings

In this applicability and reliability study, 97% of the ED visits were successfully assessed with AT-HARM10 and the IRR was substantial (Cohen’s kappa: 0.70).

### Strengths and weaknesses

This study was performed with the same assessment method as in the previous AT-HARM10 validation study [17] and in a large patient sample (n = 400), strengthening the validity of the results. However, AT-HARM10’s accuracy for ED visits was not assessed and the reliability of the results between multiple pairs of researchers was not investigated. Finally, assessments with AT-HARM10 depend on completeness of and access to patient health records, which may limit its use in other settings.

### Interpretation

Using AT-HARM10 by a final-year pharmacy student and a recent graduate to identify possible drug-related ED visits seems applicable and reliable. Wallerstedt et al. have raised common methodological issues in research on DRAs and provided suggestions for future research [11]. In line with these suggestions, we need to address that AT-HARM10 only distinguishes between visits that are *unlikely* to be and those that are *possibly* drug-related. No further causality or preventability assessment is made. The percentage of drug-related ED visits in this study (29%) seems similar to previous studies [4–6, 22]. AT-HARM10 could be used as a first step to exclude visits that are unlikely to be drug-related (71% of all ED visits in this study), followed by a more in-depth assessment by an expert panel (e.g., using causality and preventability algorithms as proposed by Leendertse et al. [23] or using the DRA adjudication guide by Thevelin et al. [16]).

### Further research

AT-HARM10 has only been validated for use in older patients in Sweden, although successfully used in middle aged patients in a Swedish pilot study [24], and adult populations (age 18 years or older) in hospitals in Norway, Denmark and the Netherlands [25–27]. To reflect the novel use of AT-HARM10 to identify drug-related ED visits as presented in this study and to improve the tool’s understandability, the tool and its instructions were revised based on feedback from other researchers that had used the tool and our own experience (Supplementary material). The first question of the tool “*Was the admission caused by an infection, or by a previously undiagnosed disease that is not drug-related?*” was rephrased to also include causes by any symptoms, signs or abnormal clinical and laboratory findings when a formal diagnosis has not yet been made. Other changes concern clarifications and examples, and a paragraph on how to use the tool as a validated method was added. Further validation studies with the updated version of AT-HARM10 and examples of the use of the tool in other settings and populations are warranted.

## Conclusion

It seems applicable and reliable to identify possible drug-related ED visits in addition to DRAs in older patients with AT-HARM10.

## Supplementary Information

Below is the link to the electronic supplementary material.

AT-HARM10 – Instructions (Updated: May 2022) (PDF 287 kb)
