# Supplementary material for: Drug-related emergency department visits in older patients: an applicability and reliability study of an existing assessment tool
Source: Int J Clin Pharm. 2022 Jul 15;44(4):1078–82. doi: 10.1007/s11096-022-01456-x (PMC9393129; doi:10.1007/s11096-022-01456-x)
Supplement: Supplementary file 1 — AT-HARM10 – Instructions (Updated: May 2022) (PDF 287 kb) [file 11096_2022_1456_MOESM1_ESM.pdf]

AT-HARM10 – Instructions

Assessment Tool for identifying Hospital Admissions Related to Medications

Introduction and definitions

The Assessment Tool for identifying Hospital Admissions Related to Medications (AT-HARM10) is a screening tool consisting of ten questions used to determine if a hospital admission may be medication-related. A medication-related admission, often referred to as drug-related admission (DRA), is a hospital admission where a drug-related problem (DRP) is either the main cause or a significantly contributing cause of admission (i.e., without the DRP, the patient would not have been admitted). DRPs are defined here as 'undesirable patient experiences that involve drug therapy and that actually or potentially interfere with desired patient outcomes' (1). These can involve not only adverse drug reactions to prescribed medication, but also problems such as inappropriate prescribing and non-compliance and problems related to over-the-counter (OTC) medications. The tool does not take account of if an admission was preventable (e.g., an admission caused by side effects of appropriate medication treatment will be considered drug-related). AT-HARM10 was developed to measure the incidence of *possible* DRAs.

How to use AT-HARM10

The user of AT-HARM10 should not have to go through all the patient data in a patient's medical records, as that would take an inordinate amount of time. The patient data that we recommend to use in the assessment (and that were used in the validation study of the tool (2)) include admission notes on the current admission, medication list, laboratory data, pharmacists' notes if available, and the discharge summary for the admission. All registered medications, including OTC medications, should be considered in the assessment. Non-registered complementary and alternative medicine products and dietary supplements need not be considered.

The tool comprises ten questions which can only be answered 'Yes' or 'No'. For further clarification of each question, please see the examples below. Questions 1–3 are used to identify admissions that are **unlikely to be drug-related** (**U**), while questions 4–10 are used to identify **possible drug-related** (**P**) admissions. The assessment is finished as soon as the answer 'Yes' is given in response to any question, resulting in the admission being either U or P. This means that it is neither necessary to answer the remaining questions when a 'Yes' answer has been given, nor necessary to answer the questions in any specific order. More than one question could be answered with 'Yes', although questions 1–3 and 4–10 should be mutually exclusive. If **all** the questions are answered 'No', the assessment is ambiguous and could perhaps be examined by an expert panel.

Please note: While the reason for *visiting the emergency department (ED)* might be non-drug-related (e.g., chest pain, headache), *the primary cause of admission* might in some cases turn out to be drug-related (e.g., low potassium levels discovered while at the ED, which were worsened by a diuretic). In these cases, the admission should be classified as P. On the other hand, if AT-HARM10 is used to identify drug-related ED visits instead of DRAs (see below), the focus of the assessment should be on the reason for the encounter (presenting complaint) rather than on the reason for admission.

The use of AT-HARM10 as a validated method

Use by student pairs with appropriate training

AT-HARM10 has been validated for use by pharmacy student pairs in Sweden and the Netherlands (2,3). In these studies, the students were in at least their fifth year of pharmacy education (MSc.). They first received a training session from the developers of the tool and/or an experienced clinical pharmacist and researcher, including test cases and case discussions. Each student then independently applied AT-HARM10, classifying admissions as either unlikely to be drug-related or possibly drug-related. After assessing admissions separately, each student pair discussed the admissions that they disagreed on to reach consensus. An experienced researcher was available if consensus could not be achieved. It is recommended to provide appropriate training and make use of a pair of student assessors (instead of a single assessor) with advanced knowledge of pharmacotherapy, to ensure the validity of the results when using AT-HARM10.

Use in older population

AT-HARM10 has been validated for use in older patients (age 65 years or older) at Swedish hospitals and in an adult population (age 18 years or older; median age: 70 years) at a hospital in the Netherlands (2,3). In both the initial validation study and the clinical trial for which AT-HARM10 was initially developed (4), patients were excluded if they received palliative treatment. However, palliative patients (n=113) were included in the Dutch study (3). Hence, AT-HARM10 is valid for use in an older adult population and should be used with caution in patients receiving palliative treatment.

Use for assessment of ED visits

AT-HARM10 was originally developed and validated to assess hospital admissions. In a recent study, the tool was applied to 184 ED visits of patients aged 65 years and older who participated in a clinical trial at Swedish hospitals (5). It was deemed applicable and reliable to use AT-HARM10 to identify possible drug-related ED visits in older patients.

Changes to the initial validated version of AT-HARM10

The first question of the tool *"Was the admission caused by an infection, or by a previously undiagnosed disease that is not drug-related?"* was rephrased to also include causes by any symptoms, signs or abnormal clinical and laboratory findings when a formal diagnosis has not yet been made (i.e., a situation that is more common in ED visits than hospital admissions due to limited availability of diagnostic data). Other changes concerned clarifications, examples and a paragraph on how to use the tool as a validated method was added.

References

1) Strand L, Morely P, Cipolle R, et al. Drug-related problems: their structure and function. *Ann Pharmacother*. 1990; https://doi.org/10.1177/106002809002401114
2) Kempen TGH, Hedström M, Olsson H, et al. Assessment tool for hospital admissions related to medications: development and validation in older patients. *Int J Clin Pharm*. 2019; https://doi.org/10.1007/s11096-018-0768-8
3) Coppes, T, van der Kloes, J, Dalleur, O, et al. Identifying medication-related readmissions: Two students using tools vs a multidisciplinary panel. *Int J Clin Pract*. 2021; https://doi.org/10.1111/ijcp.14768
4) Kempen TGH, Bertilsson M, Hadziosmanovic N, et al. Effects of hospital-based comprehensive medication reviews including postdischarge follow-up on older patients' use of health care: a cluster randomized clinical trial. *JAMA Netw Open*. 2021; https://doi.org/10.1001/jamanetworkopen.2021.6303
5) Kempen TGH, Hedman A, Gillespie U. Drug-related emergency department visits in older patients: an applicability and reliability study of an existing assessment tool. *Int J Clin Pharm*. 2022; submitted manuscript.

AT-HARM10

Assessment Tool for identifying Hospital Admissions Related to Medications

Note: Questions 1–3 are used to identify admissions unlikely to be drug-related, while questions 4–10 are used to identify possible drug-related admissions (DRAs). The assessment is finished as soon as the answer 'Yes' is given for any question → U (unlikely to be drug-related) or P (possibly drug-related). If all the questions are answered 'No', the assessment is ambiguous.

1. Was the admission caused by an *infection,* a previously *undiagnosed* disease (e.g., diabetes or heart failure), or symptoms, signs and abnormal clinical and laboratory findings (if no specific diagnosis has been made) that do *not* seem to be *drug-related*?

Yes → U (unlikely to be drug-related)

No → NQ (next question)

Note: If historical information is used for the assessment and this information (e.g., previous laboratory findings) already points towards a disease or diagnosis that may have been undertreated or sub-optimally treated, this may be a *possible* DRA (question 8).

2. Was the admission caused by progression of a previously diagnosed disease that is *not drug-related*?

Yes → U

No → NQ

Note: Appropriateness of medication treatment should only be considered in relation to this question to determine whether the admission is primarily caused by disease progression (*unlikely* DRA) or by suboptimal medication treatment or use (*possible* DRA, questions 4–10).

Note: With the progression of some chronic diseases, such as congestive heart failure or diabetes, a drug-related component can rarely be excluded.

3. Was the admission caused by physical trauma, substance intoxication, social circumstances or allergies (e.g., car accident, alcohol, mushroom poisoning, wasp allergy) that was/were *not drug-related*?

Yes → U

No → NQ

4. Is it hinted or stated in the medical records that the admission was *drug-related* (including non-compliance)?
Yes → P (possibly drug-related)
No → NQ

5. Might (side) effects of the medications the patient was taking (prescribed or non-prescribed) prior to hospitalisation have caused the admission (including over-treatment)?
Yes → P
No →NQ
Note: An admission caused by side effects of appropriate medication treatment should be classified as *possibly* drug-related.

6. Are there abnormal laboratory results or vital signs that could be *drug-related* and might have caused the admission?
Yes → P
No → NQ

7. Was there any drug-drug interaction or drug-disease interaction (i.e., a contraindication) that might have caused the admission?
Yes → P
No → NQ

8. Did the patient have any *previously* diagnosed, untreated or sub-optimally treated (e.g., dose too low) indications that might have caused the admission?
Yes → P
No → NQ

9. Was the patient admitted because of a problem with the dosage form or pharmaceutical formulation (e.g., failure to receive the medication)?
Yes → P
No → NQ

10. Is the cause of the admission a response to cessation or withdrawal of medication treatment?
Yes → P
No → P (the tool has not been able to rule out that the admission was drug-related)

AT-HARM10 – Examples

Assessment Tool for identifying Hospital Admissions Related to Medications

Representative examples of when a question should be answered 'Yes' or 'No'.

1. Was the admission caused by an *infection,* a previously *undiagnosed* disease (e.g., diabetes or heart failure) or symptoms, signs and abnormal clinical and laboratory findings (if no specific diagnosis has been made) that do *not* seem to be *drug-related*?

   Note: If historical information is used for the assessment and this information (e.g., previous laboratory findings) already points towards a disease or diagnosis that may have been undertreated or sub-optimally treated, this may be a *possible* DRA (question 8).

   Yes: A patient admitted because of pneumonia that was *not related* to the patient's medications.

   Yes: A patient admitted because of rectal bleeding which, after investigation, was found to have been caused by a tumour.

   Yes: A patient admitted with unclear diagnosis and new symptoms that cannot be explained by the patient's current medications.

   No: A patient receiving immunosuppressive treatment who is admitted with infection.

   No: A patient admitted with new symptoms indicating heart failure (oedema, shortness of breath) and a history of excessive use of non-steroidal anti-inflammatory drugs (NSAIDs).

2. Was the admission caused by progression of a previously diagnosed disease that is *not drug-related*?

   Note: Appropriateness of medication treatment should only be considered in relation to this question to determine whether the admission is primarily caused by disease progression (*unlikely* DRA) or suboptimal medication treatment or use (*possible* DRA, questions 4–10).

   Note: With the progression of some chronic diseases, such as congestive heart failure or diabetes, a drug-related component can rarely be excluded).

   Yes: A patient admitted because of progression of cancer that is not related to the patient's medications.

   Yes: A patient admitted because of exacerbation of congestive heart failure, which worsened despite optimal treatment (the medication treatment seems to follow the applicable treatment guidelines), with no signs of non-compliance.

   No: A diabetic patient admitted because of hyperglycaemia without another reason for admission (hyperglycaemia should never lead to admission in a patient who is optimally treated).

3. Was the admission caused by physical trauma, substance intoxication, social circumstances or allergies (e.g., car accident, alcohol, mushroom poisoning, wasp allergy) that was/were *not drug-related*?

Yes: A patient admitted because of alcohol intoxication or a car accident that was *not related* to the patient's *medication* use.

No: A patient admitted because of alcohol intoxication worsened by concomitant use of sedatives.

---

4. Is it hinted or stated in the medical records that the admission is *drug-related* (including non-compliance)?

Yes: A physician states in the discharge note that the patient was admitted because of constipation caused by a lack of laxative treatment during treatment with a strong opioid.

Yes: A patient admitted because of an epileptic seizure and a note in the medical records that the patient is known to be non-compliant.

No: A physician states upon admission that the admission may have been caused by medication, but an alternative non-drug-related cause is identified later during the hospital admission.

5. Might (side) effects of the medications the patient was taking (prescribed or non-prescribed) prior to hospitalisation have caused the admission (including over-treatment)?

Note: An admission caused by side effects of appropriate medication treatment should be classified as *possibly* drug-related.

Yes: A patient admitted with gastric bleeding who uses acetylsalicylic acid to prevent thrombotic events (regardless of if there is a correct indication and of the use of a proton pump inhibitor for gastric protection).

Yes: A patient admitted because of lactic acidosis after continuing medication with metformin while experiencing dehydrating stomach flu.

Yes: A patient who uses antihypertensive medication and is admitted due to a fall caused by orthostatic hypotension.

No: During hospital admission, a patient complains about long-term hoarseness caused by bronchodilators for chronic obstructive pulmonary disease (COPD), but the main reason for admission was infection-related diarrhoea and dehydration.

6. Are there abnormal laboratory results or vital signs that could be *drug-related* and might have caused the admission?

   Yes: A patient admitted with a serum digoxin concentration of 3.4 nmol/L (toxic concentration) which may have been the cause of admission.

   Yes: A patient admitted because of hypokalaemia (s-potassium < 3.5 mmol/L) and prescribed a diuretic.

   Yes: A patient with epilepsy admitted with seizures and prescribed a seemingly adequate dose of carbamazepine, but the plasma concentration measured is too low.

   No: An s-potassium of 3.3 mmol/L is discovered during admission and the patient has been using a diuretic, but the reason for admission was gastric bleeding that was not drug-related.

7. Was there any *drug-drug interaction* or *drug-disease interaction* (i.e., a contraindication) that might have caused the admission?

   Yes: A patient who was taking diclofenac and warfarin in combination before admission, admitted because of gastrointestinal bleeding.

   Yes: A patient who was taking tramadol, citalopram and mirtazapine, admitted because of serotonin syndrome.

   Yes: A patient, previously diagnosed with bilateral renal artery stenosis, admitted because of acute renal failure after taking an angiotensin-converting enzyme (ACE) inhibitor.

   Yes: A patient with dementia, who has recently been prescribed an anticholinergic medication (e.g., hydroxyzine), admitted with confusion.

   No: A patient gets prescribed a proton pump inhibitor during hospital admission because of a previously missed interaction between diclofenac and apixaban, but the reason for admission was an ankle fracture.

8. Did the patient have any *previously* diagnosed, untreated or sub-optimally treated (e.g., dose too low) indications that might have caused the admission?

   Yes: A patient diagnosed with congestive heart failure, who was taking only a starting dose of ACE inhibitor (unjustifiably low dose), admitted because of fluid retention and dyspnoea.

   Yes: A patient who had a prior diagnosis of osteoporosis but was not taking osteoporosis prophylaxis, admitted because of a hip fracture.

   No: A patient recently diagnosed with congestive heart failure, who was taking adequate doses of an ACE inhibitor, a beta blocker and a mineralocorticoid receptor antagonist, and furosemide as needed, admitted because of fluid retention and dyspnoea.

9. Was the patient admitted because of a problem with the dosage form or pharmaceutical formulation (e.g., failure to receive the medication)?

Yes: A patient admitted with worsening asthma who was found to be unable to use their inhalers correctly.

Yes: A patient admitted with palpitations who was found to be unable to swallow tablets and had been crushing slow-release antihypertensive tablets, which had to be swallowed whole to retain their slow-release effect.

No: A patient admitted with palpitations who was found to be unable to swallow tablets and had been crushing slow-release iron supplementation tablets, which had to be swallowed whole to retain their slow-release effect.

10. Is the cause of the admission a response to cessation or withdrawal of medication treatment?

Yes: A patient whose prednisolone treatment was discontinued too abruptly, admitted with nausea, vomiting and diarrhoea.

No: A patient whose prednisolone treatment was discontinued a few months earlier, admitted with nausea, vomiting and diarrhoea caused by an infection.
